# Value of the NF-κB signalling pathway and the DNA repair gene PARP1 in predicting distant metastasis after breast cancer surgery

**DOI:** 10.1038/s41598-023-49156-4

**Published:** 2024-02-22

**Authors:** Kaiyong Pan, Xiabin Li, Junfang He, Yuxi Lei, Yongxin Yang, Deyong Jiang, Yan Tang

**Affiliations:** 1 School of Public Health, Southwest Medical University, 1 Xianglin Road, Luzhou, 646000 Sichuan China; 2 Department of Pathology, The First Affiliated Hospital of Southwest Medical University, 25 Taiping Road, Luzhou, 646000 Sichuan China; 3 Guizhou QianNan People’s Hospital, 9 Enfeng Road, Duyun, 558099 Guizhou China; 4 Sichuan Luzhou Center for Disease Control, 31 Datong Road, Luzhou, 646000 Sichuan China

**Keywords:** Cancer, Risk factors, Breast cancer

## Abstract

The DNA repair gene PARP1 and NF-κB signalling pathway affect the metastasis of breast cancer by influencing the drug resistance of cancer cells. Therefore, this study focused on the value of the DNA repair gene PARP1 and NF-κB pathway proteins in predicting the postoperative metastasis of breast cancer. A nested case‒control study was performed. Immunohistochemical methods were used to detect the expression of these genes in patients. ROC curves were used to analyse the predictive effect of these factors on distant metastasis. The COX model was used to evaluate the effects of PARP1 and TNF-α on distant metastasis. The results showed that the expression levels of PARP1, IKKβ, p50, p65 and TNF-α were significantly increased in the metastasis group (*P* < 0.001). PARP1 was correlated with IKKβ, p50, p65 and TNF-α proteins (*P* < 0.001). There was a correlation between IKKβ, p50, p65 and TNF-α proteins (*P* < 0.001). ROC curve analysis showed that immunohistochemical scores for PARP1 of > 6, IKKβ of > 4, p65 of > 4, p50 of > 2, and TNF-α of > 4 had value in predicting distant metastasis (*Se*_PARP1_ = 78.35%, *Sp*_PARP1_ = 79.38%, *AUC*_PARP1_ = 0.843; *Se*_p50_ = 64.95%, *Sp*_p50_ = 70.10%, *AUC*_p50_ = 0.709; *Se*_TNF-α_ = 60.82%, *Sp*_TNF-α_ = 69.07%, *AUC*_TNF-α_ = 0.6884). Cox regression analysis showed that high expression levels of PARP1 and TNF-α were a risk factor for distant metastasis after breast cancer surgery (*RR*_PARP1_ = 4.092, 95% *CI* 2.475–6.766, *P* < 0.001; *RR*_TNF-α_ = 1.825, 95% *CI* 1.189–2.799, *P* = 0.006). Taken together, PARP1 > 6, p50 > 2, and TNF-α > 4 have a certain value in predicting breast cancer metastasis, and the predictive value is better when they are combined for diagnosis (*Se*_combine_ = 97.94%, *Sp*_combine_ = 71.13%).

## Introduction

The most prevalent form of malignant tumour worldwide, particularly in women, is breast cancer (BC), which is also the most common cause of death from tumours in women^1,2^. In recent years, many studies have suggested that tumour metastasis is the primary cause of BC patient death, and prognosis is directly correlated with tumour metastasis^3^.

According to the findings, the postoperative metastasis of BC is not only related to the age of patients, the expression of ER, Ki67, PR, P53, HER2, and Ecad, the status of axillary lymph nodes, tumour size, and histological stage and grade but also closely related to DNA repair genes. The functions of the DNA repair gene PARP1 include not only DNA damage repair and genome stabilization^4^, cell signal transduction and transcriptional regulation of various proinflammatory factors but also the proliferation, differentiation, apoptosis and occurrence and development of tumour cells^5^. In BC, poor prognosis has been correlated with the upregulation of PARP1^6^. Studies have shown that abnormal expression of PARP1 is closely related to lymph node metastasis in BC, and those with high expression levels of PARP1 have a shorter survival time^7^. A study showed that PARP1 is a risk factor for BC metastasis^7^. IKK/NF-κB is an important signalling pathway for tumour development and immune and inflammatory responses. It is one of the regulatory signalling pathways of PARP1. A crucial component of the NF-κB transcription complex is PARP1. NF-κB-mediated gene transcription can be prevented by inhibiting PARP1 enzyme activity. Moreover, PARP1 functions in NF-κB-mediated transcription as a coactivator. Studies show that NF-κB is expressed more aggressively in a number of cancers with an epithelial origin and that structurally activated NF-κB is closely associated with epithelial-derived tumours, such as squamous cell carcinoma^8^, colorectal cancer^9^, BC^10^ and renal cell carcinoma^11^. In BC, inappropriate expression of the NF-κB protein has been linked to abnormal expression of proteins relevant to the epithelial stroma^12,13^. NF-κB regulates the development, spread and invasion of BC cells. It may also promote the growth of BC into a phenotype with a negative oestrogen receptor, with an increased capacity for invasion and a higher histological grade^14^. Tumour necrosis factor-α (TNF-α), one of the several downstream proteins of the IKK/NF-κB signalling pathway, is a prominent inflammatory factor in the inflammatory environment, and it is strongly linked to cancer diagnosis and prognosis^15^. Studies indicate that BC patients have considerably higher serum levels of TNF-α than healthy people and that these concentrations are correlated with tumour invasion and TNM staging^16,17^. Additionally, it has been found that the expression of TNF-α can be an invaluable indicator for assessing overall survival and progression-free survival in patients with BC metastasis^18^, and the use of exogenous TNF-α can promote clinically harmful processes such as the invasion and migration of BC cells^19–21^.

The most prevalent category of cancer caused by inflammation is BC. PARP1^22,23^, IKK/NF-κB^8–11^ and TNF-α^24–28^ are all related to the metastasis and severity of BC. In addition, PARP1 and TNF-α^29^, PARP1 and IKK/NF-κB, and IKK/NF-κB and TNF-α^30–32^ have regulatory effects in tumours that influence tumour occurrence and development. Therefore, this study focused on the value of the DNA repair gene PARP1 and NF-κB pathway proteins in predicting postoperative metastasis of BC.

## Materials and methods

### Sample

#### Subjects

The BC follow-up cohort established at the Affiliated Hospital of Southwest Medical University in 2014 was the study object. The smallest follow-up term was 4 months, the longest was 59 months, and the average follow-up period was 31.71 months. (1) The inclusion criteria were as follows: 1. individuals with newly discovered BC who received surgery; 2. individuals with complete personal information and medical records; 3. availability of complete and reliable paraffin block samples; and 4. voluntary participation. (2) The exclusion criteria were as follows: 1. nonprimary BC patients and patients with other serious underlying diseases; 2. people with mental illness and dying patients; and 3. patients with other cancers or a history of other tumours.

#### Study object grouping

Depending on whether the patient's remote transfer occurred after surgery, the patients were divided into a control group and a metastatic group, with 97 patients in each group. Patients who had metastasis from distant metastasis during the follow-up process were designated the metastatic group. According to the 1:1 matching principle, patients without metastases in the same time period were designated the control group. Age (3 years), surgery time (less than 1 month), and surgical method alignment with other surgical treatments were the matching criteria.

#### Ethical review

The ethical details were as follows: (1) All studies were agreed by patients, and patients gave their informed consent to participate. (2) The Southwest Medical University's Biomedical Ethics Committee has examined and approved the research design.This study meets the clinical research ethical requirements. (3) Ethics review application number: XNYD2018001. We confirm that all the experiment protocol for involving humans was in accordance with the guidelines of national in the manuscript.

### Detection of indicators

The clinical pathological data and tissue slices were obtained from the Breast Surgery and Pathological Department of the Affiliated Hospital of Southwest Medical University. The paraffin block samples were 100% tumour cells (all determined and diagnosed by pathologists). Three-millimetre paraffin sections were dried, deparaffinized, and rehydrated across a solution gradient from alcohol to water. Pressure cooker treatment in EDTA buffer for 10 min was used to achieve heat-mediated antigen retrieval (pH 9.0). Primary mouse anti-human monoclonal antibodies against PARP1, IKKβ, p65, p50, and TNF-α were applied to the slides and incubated for 120 min at 25 °C (Dako, DK). After being washed, the sections were incubated for 30 min at 25 °C with the secondary antibody (Envision, HRP rabbit/mouse, DK). We obtained negative controls by omitting the main antibody. DAB was used to visualize the slides.

Japan Olympus Co., Ltd. microscopes were used in the examination, observation and mapping by experienced pathologists. The expression levels of PARP1, IKKβ, p50, p65 and TNF-α were detected in tumour cells. Five fields of view were randomly selected under 200 × magnification for statistical counting. The staining and percentage of positive tumour cells were used as grading criteria. Positive cell staining scores were as follows: no DAB staining, 0 points; light brown-yellow, 1 point; brown-yellow, 2 points; and deep brown-yellow, 3 points. The percentage was divided into 5 semiquantitative levels: 0, 0–5%; 1, 6–25%; 2, 26–50%; 3, 51–75%; and 4 > 75%. The statistical score = the staining score x the staining percentage.

### Data analysis

The data obtained in this experiment were entered into Epidata3.1 software by multiple people and rechecked. SPSS 26.0 Statistics Software was used to perform statistical analysis of the data. Data that did not meet a normal distribution were represented by the median and interquartile range ($Median\;(IQR)$), and the Wilcoxon rank-sum test was used to assess comparisons between groups. The correlation analysis was analysed by Spearman grade correlation. The multifactor analysis used the Cox risk model. ROC analysis was used to predict associated proteins that are related to the prediction of BC metastasis, and *P* < 0.05 was considered statistically significant.

## Results

### Pathological data characteristics of BC patients

The findings revealed that there were significant differences between the two groups in terms of PR and lymph node metastases (*P* < 0.05), as shown in Table 1.

**Table 1:** The distribution of clinical and pathological characteristics in BC patients (n(%)).

Clinical pathogenic parameters	n	Nonmetastasis	Metastasis	$\chi^{2}$	*P*
Age					
0~	91	43 (44.33)	48 (49.48)	0.517	0.472
50~	103	54 (55.67)	49 (50.52)		
ER					
−	75	31 (31.96)	44 (45.36)	3.674	0.055
+	119	66 (68.04)	53 (54.64)		
PR					
−	102	44 (45.36)	58 (59.79)	4.052	0.044
+	92	53 (54.64)	39 (40.21)		
HER2					
−/+	123	63 (64.95)	60 (61.86)	0.200	0.655
+++	71	34 (35.05)	37 (38.14)		
E-Cad					
−	22	13 (13.40)	9 (10.23)	0.820	0.365
+	172	84 (85.60)	88 (89.77)		
P53					
−	100	53 (54.64)	47 (48.45)	0.743	0.389
+	94	44 (45.36)	50 (51.55)		
Ki67					
0~	50	28 (28.87)	22 (22.68)	0.970	0.325
20~	144	69 (71.13)	75 (77.32)		
Molecular					
Luminal A	34	21 (21.65)	13 (13.40)	4.386	0.356
Luminal B	51	26 (26.80)	25 (25.77)		
Luminal HER2	43	23 (23.71)	20 (20.62)		
HER2-enriched	23	10 (10.31)	13 (13.40)		
Basal-like	43	17 (17.53)	26 (26.80)		
Number of lymph node metastases					
0	65	45 (46.39)	20 (20.62)	24.806	< 0.001
1~	41	25 (25.77)	16 (16.49)		
4~	37	12 (12.37)	25 (25.77)		
10~	51	15 (15.46)	36 (37.11)		
Tissue pathology					
In situ cancer	11	7 (7.22)	4 (4.12)	0.867	0.352
Infiltrated nonspecific BC	183	90 (92.78)	93 (95.88)		
Size of the lump (cm)					
0~	52	29 (29.90)	23 (23.71)	2.170	0.338
2~	116	53 (54.64)	63 (64.95)		
5~	26	15 (15.46)	11 (11.34)		
WHO level					
I	6	5 (5.15)	1 (1.03)	3.206	0.201
II	121	57 (58.76)	64 (65.98)		
III	67	35 (36.08)	32 (32.99)		

### Protein expression of PARP1 and NF-κB in the two groups of BC patients

#### Expression of PARP1 in the two groups

**Figure 1:**
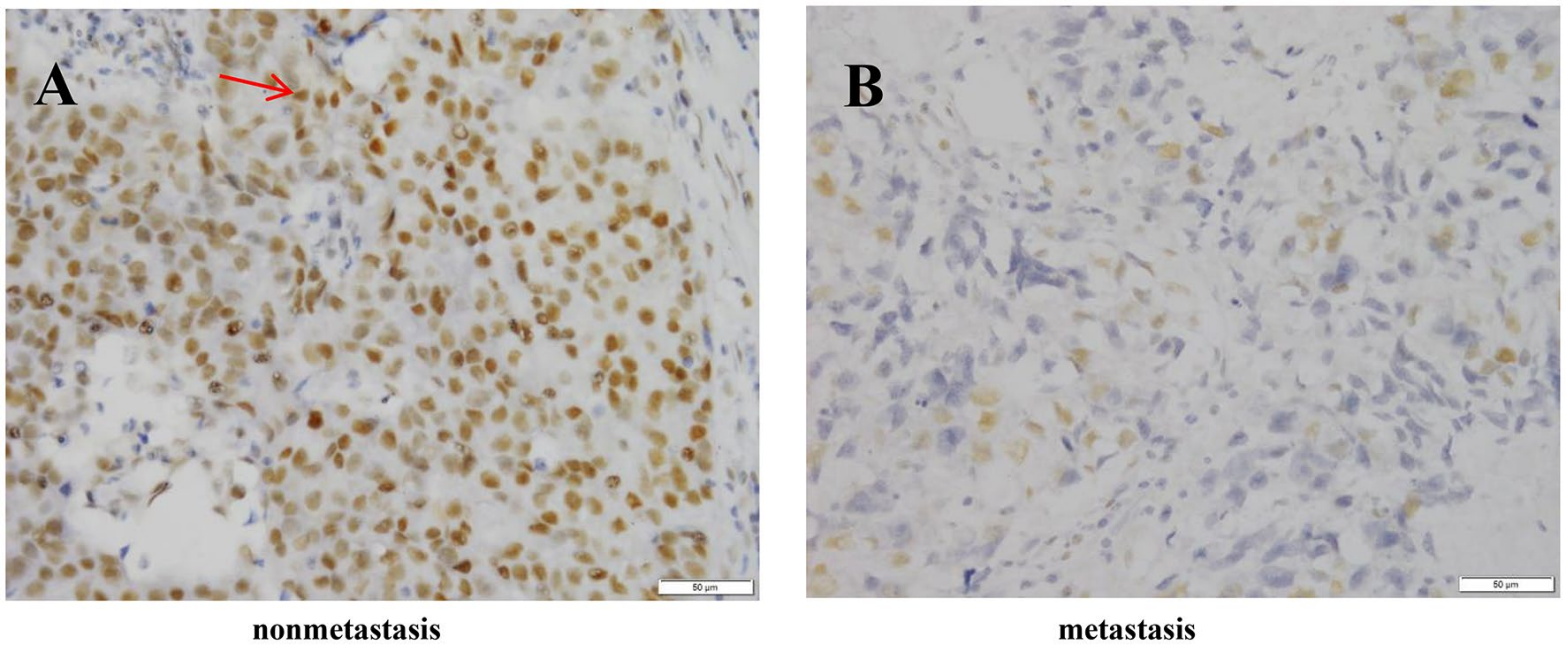
PARP1 expression in the two groups (200 ×). (**A**) PARP1 was strongly expressed and deeply stained in metastatic breast cancer tissues; (**B**) PARP1 was underexpressed and lightly stained in nonmetastatic breast cancer tissues.

**Figure 2:**
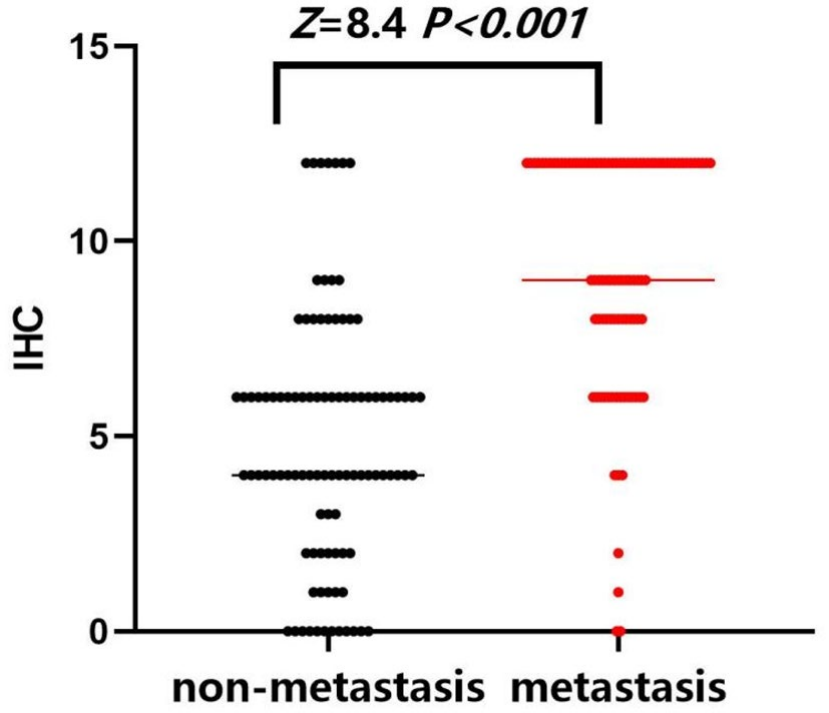
Distribution of PARP1 expression in the two groups. PARP1’s median immunohistochemical score for distant metastases after BC was 9.0, and the quarter distance was 4.0 (9, 12); the median immunohistochemical score of PARP1 in the group without metastasis after BC surgery was 4.0, and the interquartile interval was 3.5 (2.5, 6).

The PARP1 protein was brown‒yellow in cells and was observed in both the cytoplasm and nucleus, but it was mainly expressed in the cytoplasm, as shown in Fig. 1. In Fig. 2, compared to the nonmetastatic group, the metastatic group had higher expression levels (*Z* = 8.4, *P* < 0.001).

#### Expression of NF-κB proteins in the two groups

**Figure 3:**
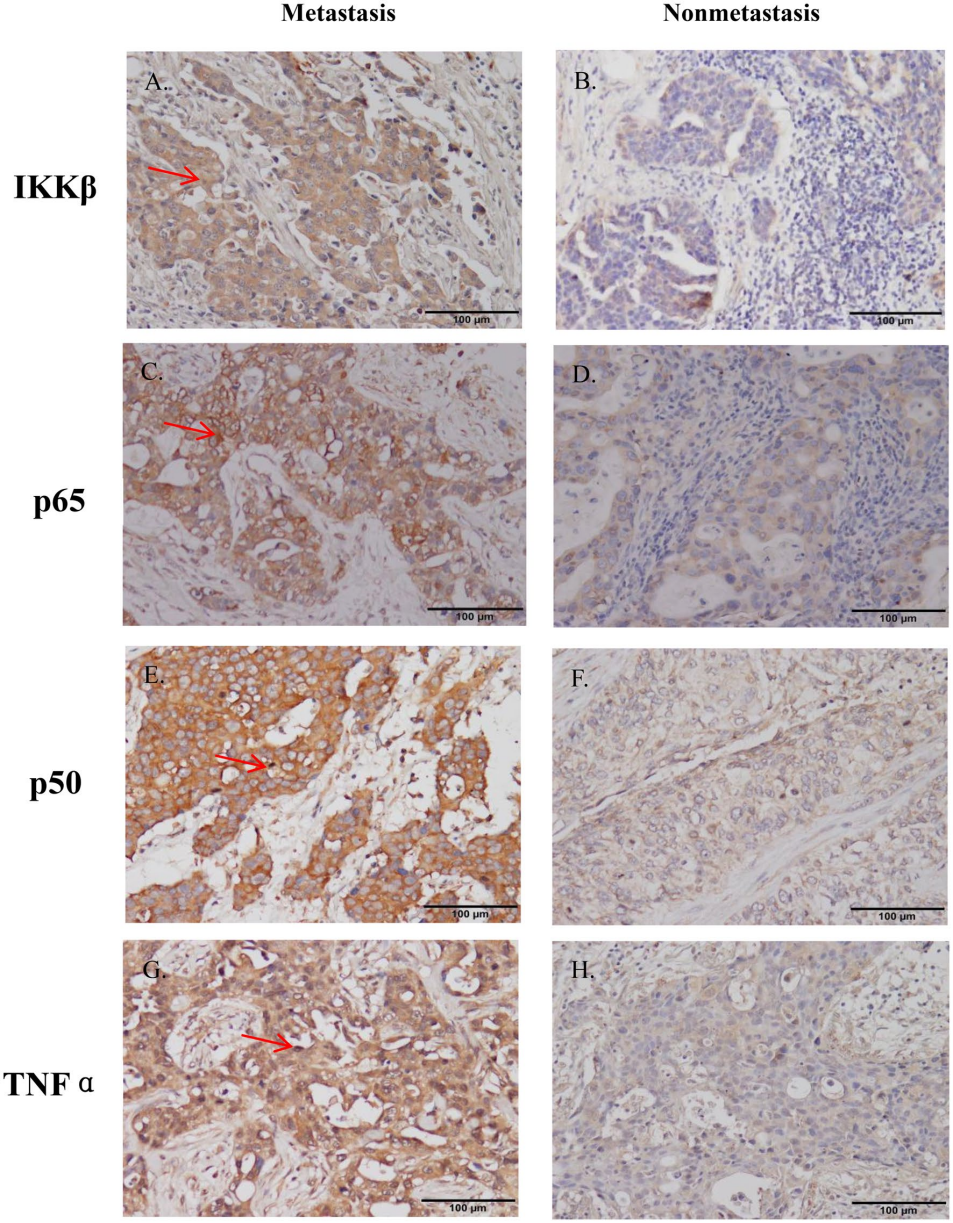
Immunohistochemistry of NF-κB proteins in the two groups. (**A**) IKKβ was strongly expressed and deeply stained in metastatic breast cancer tissues; (**B**) IKKβ was underexpressed and lightly stained in nonmetastatic breast cancer tissues; (**C**) p65 was strongly expressed and deeply stained in metastatic breast cancer tissues; (**D**) p65 was underexpressed and lightly stained in nonmetastatic breast cancer tissues; (**E**) p50 was strongly expressed and deeply stained in metastatic breast cancer tissues; (**F**) p50 was underexpressed and lightly stained in nonmetastatic breast cancer tissues; (**G**) TNFα strongly expressed and deeply stained in metastatic breast cancer tissues; (**H**) TNFα was underexpressed and lightly stained in nonmetastatic breast cancer tissues.

**Figure 4:**
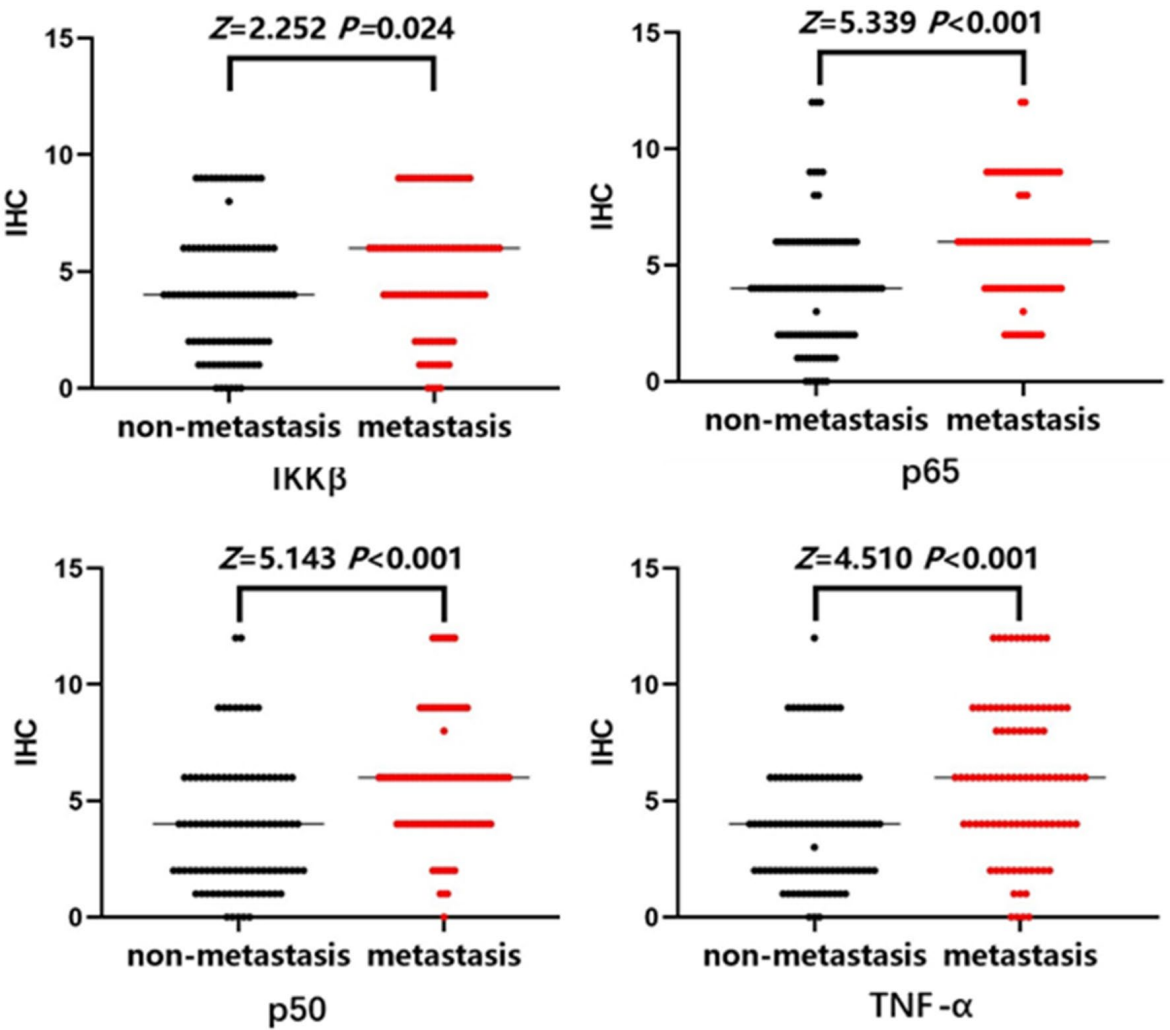
Expression of NF-κB proteins in the two groups.

Based on the results, TNF-α, IKKβ, p65 and p50 proteins were expressed in the cytoplasm of BC cells, and their staining was brownish-yellow (Fig. 3). In paraffin blocks, the expression levels of IKKβ, p65, p50, and TNF-α proteins were significantly greater in the metastasis group than in the nonmetastasis group (*P* < 0.05) (Fig. 4).

### Impact of PARP1 and NF-κB proteins on distant metastasis

#### Correlation between PARP1 and NF-κB proteins in distant metastasis after BC surgery

Through Spearman correlation analysis, the expression of PARP1 was positively correlated with that of IKKβ (*r* = 0.163, *P* < 0.001); the expression of IKKβ was positively correlated with that of p65 (*r* = 0.392, *P* < 0.001) and p50 (*r* = 0.406, *P* < 0.001); the expression levels between p50 and p65 were also positively correlated (*r* = 0.486, *P* < 0.001); and TNF-α expression was positively correlated with the expression of p65 (*r* = 0.558, *P* < 0.001) and p50 (*r* = 0.510, *P* < 0.001). The expression of TNF-α was positively correlated with BC (*r* = 0.281, *P* < 0.001) (Table 2).

**Table 2:** Correlation of PARP1 and NF-κB proteins.

Variable	Metastasis	PARP1	p50	p65	TNF-α	IKKβ
Metastases	1	0.605**	0.370**	0.384**	0.325**	0.162*
PARP1	–	1	0.200**	0.208**	0.311**	0.163*
p50	–	–	1	0.486**	0.510**	0.406**
p65	–	–	–	1	0.558**	0.392**
TNF-α	–	–	–	–	1	0.466**
IKKβ	–	–	–	–	–	1

**P < 0.001.

#### Multifactor analysis of PARP1 and TNF-α on distant metastases after BC

##### Selection of candidate variables in the Cox regression model

**Figure 5:**
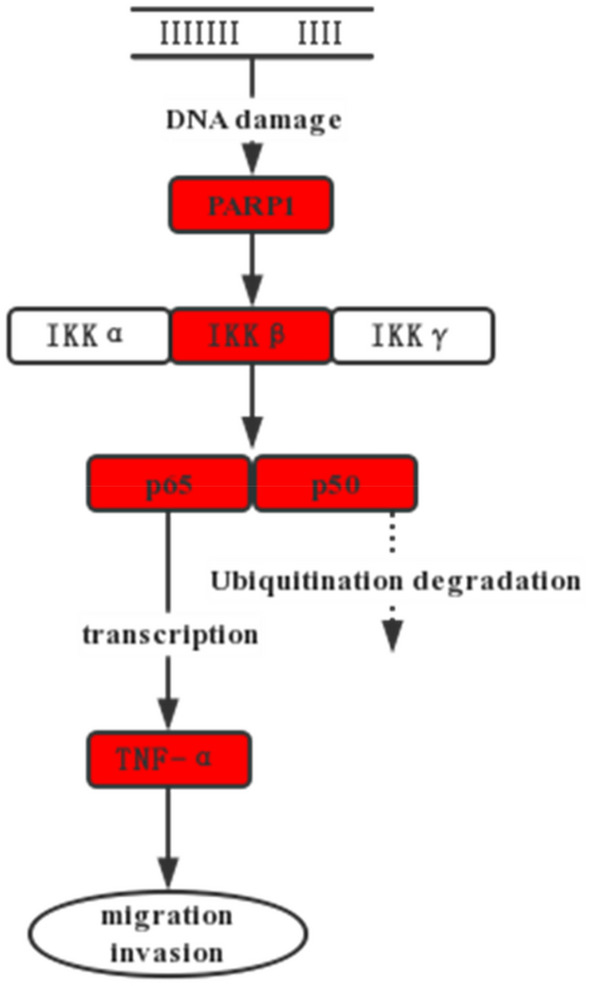
Regulatory relationship between PARP1 and NF-κB proteins.

**Figure 6:**
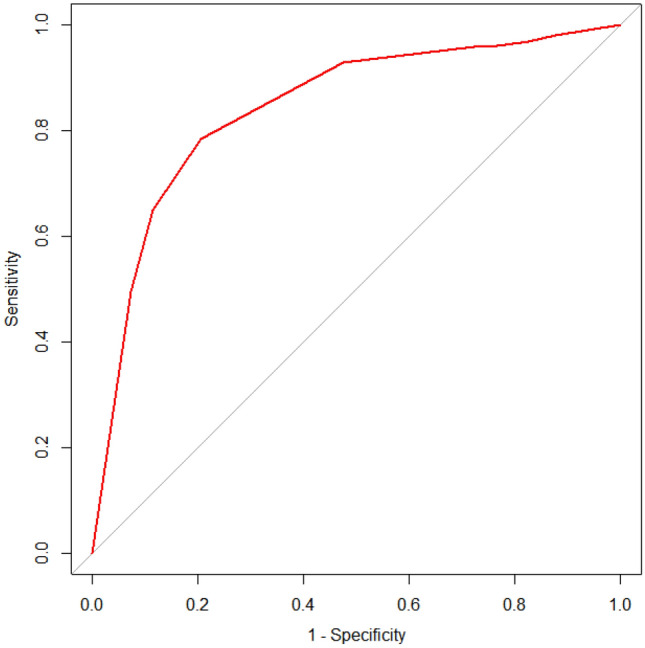
Predictive value of PARP1 in BC metastasis.

In univariate analysis, the expression of PR, lymph node metastasis, PARP1, and NF-κB proteins was associated with distant metastasis of BC (*P* < 0.05). There was a certain regulatory relationship between the PARP1 gene and the NF-κB pathway, and PARP1 regulated the expression of IKKβ, p65, p50 and TNF-α (Fig. 5). Spearman correlation analysis showed that PARP1 was correlated with p50, p65, TNF-α and IKKβ, as shown in the correlation matrix in Table 3. Thus, the collinearity between them was taken into account, and Cox analysis combining PARP1, p50, p65, TNF-α and IKKβ could not be performed. PARP1 and TNF-α were evaluated by Cox analysis after excluding other variables that affected each other (Figs. 5 and 6).

**Table 3:** Cox regression assignment tables.

Variable	Assignment
PR	0 = negative; 1 = positive
Number of lymph nodes	0 = 0; 1 = 1–3; 2 = 4–9; 3 = ≥ 10
Outcome	0 = nonmetastasis; 1 = metastasis

##### Cox regression analysis of the influence of the PARP1 protein score on distant metastasis after BC surgery

With the status of distant metastasis of BC as the dependent variable, meaningful (*P* < 0.05) PR, number of lymph node metastases and PARP1 score in univariate analysis were taken as independent variables, and specific variables were assigned for Cox regression risk model analysis. The results showed that PARP1 (*RR* = 1.206, 95% *CI* 1.125–1.293; *P* < 0.001) and the number of lymph nodes ≥ 10 (*RR* = 2.131, 95% *CI* 1.180–3.851; *P* = 0.012) were both risk factors for distant metastasis (Table 4).

**Table 4:** Predictive role of the PARP1 score in BC metastasis.

Variable	*B*	*SE*	*Wald*	*P*	*RR*	*95% CI*
PR	0.007	0.214	0.001	0.973	0.993	0.653–1.510
PARP1	0.187	0.035	28.046	<0.001	1.206	1.125–1.293
Number of lymph nodes						
0	–	–	–	–	–	–
1~	0.269	0.340	0.629	0.428	1.309	0.673–2.548
4~	0.597	0.315	3.601	0.058	1.817	0.981–3.368
≥ 10	0.757	0.302	6.285	0.012	2.131	1.180–3.851

##### Cox regression analysis of the influence of the TNF-α protein score on distant metastasis after BC surgery

According to the Cox regression analysis model, the occurrence of postoperative distant metastasis of BC was regarded as the dependent variable, and indices such as PR, lymph node metastasis grade and TNF-α score in the univariate analysis were regarded as independent variables. The specific variables were assigned (Table 5) and then included in the Cox regression risk model. The results showed that TNF-α (*RR* = 1.123, 95% *CI* 1.052–1.199; *P* < 0.001), a number of lymph node metastases of 4–9 (*RR* = 2.763, 95% *CI* 1.512–5.048; *P* = 0.001), and a number of lymph nodes ≥ 10 (*RR* = 3.088, 95% *CI* 1.756–5.432; *P* < 0.001) were risk factors for distant metastasis after BC (Table 6).

**Table 5:** Regression assignment tables.

Variable	Assignment
PR	0 = negative; 1 = positive
Number of lymph nodes	0 = 0; 1 = 1–3; 2 = 4–9; 3 = ≥ 10
Outcome	0 = nonmetastasis; 1 = metastasis

**Table 6:** Predictive role of the TNF-α score in BC metastasis.

Variable	*B*	*SE*	*Wald*	*P*	*RR*	*95% CI*	
PR	− 0.123	0.213	0.336	0.562	0.884	0.583–1.341	
TNF-α	0.116	0.033	12.255	<0.001	1.123	1.052–1.199	
Lymph node metastasis							
0	–	–	–	–	–	–	–
1~	0.414	0.338	1.500	0.221	1.513	0.780–2.933	
4~	1.016	0.308	10.919	0.001	2.763	1.512–5.048	
≥ 10	1.128	0.288	15.319	<0.001	3.088	1.756–5.432	

### Predictive effect of PARP1 and NF-κB pathway-related proteins on distant metastases after BC

The immunohistochemical scores of PARP1, IKKβ, p65, p50, and TNF-α in the transfer group were determined. These scores are discontinuous values, such as 0–4, 6, 8, 9, and 12, but the level of protein expression did not represent a critical value for an accurate diagnosis. The effect of the return analysis of the COX multifactor was not good. Therefore, the ROC curve was used for prediction analysis and determining the cut-off values.

#### Value of PARP1 in predicting distant metastasis after BC surgery

##### ROC analysis of PARP1 for postoperative distant metastasis of BC

ROC analysis showed that PARP1 was the best factor for predicting BC metastasis when the immunohistochemical score was > 6, *Se* = 78.35%, *Sp* = 79.38%, and *YI* = 0.578 (*AUC* = 0.843, *P* < 0.001), as shown in Fig. 6, Table 7.

**Table 7:** ROC projected PARP1 cut-off values (%).

Cut-off	*Se*	*Sp*	*YI*
≥ 0	100.00	0.00	0
> 0	97.94	12.37	0.1031
> 1	96.91	17.53	0.1443
> 2	95.88	24.74	0.2062
> 3	95.88	27.84	0.2371
> 4	92.78	52.58	0.4536
> 6	78.35	79.38	0.5773
> 8	64.95	88.66	0.5361
> 9	49.48	92.78	0.4227
≥ 12	0.00	100.00	0

##### Relationship between PARP1 expression and BC metastasis

According to the cut-off value, an immunohistochemical score for PARP1 of > 6 was classified as high expression, and an immunohistochemical score for PARP1 of ≤ 6 was classified as low expression. When the two groups were compared, it was found that the high expression rate in the metastatic group was significantly higher than that in the nonmetastatic group ($\chi^{2}$ = 64.667,* P* < 0.001), as shown in Table 8.

**Table 8:** The expression of PARP1 in BC patients.

Group	Low expression	High expression	$\chi^{2}$	*P*
Nonmetastasis	77 (79.4)	20 (20.6)	64.667	< 0.001
Metastasis	21 (21.6)	76 (78.4)		

##### COX regression analysis of the influence of PARP1 protein expression on distant metastasis after BC surgery

According to the cut-off value of PARP1, the patients were divided into high- and low-expression groups. After ROC curve prediction, the variable distribution is shown in Table 9. Cox regression analysis was used to verify the predictive effect of PARP1 on postoperative metastases of BC. The results showed that the risk of mammary cancer metastasis in the high PARP1 expression group was 4.092 times that in the low PARP1 expression group (*RR* = 4.092, 95% *CI* 2.475–6.766, *P* < 0.001) (Table 10).

**Table 9:** Regression assignment tables.

Variable	Assignment
PR	0 = negative; 1 = positive
Number of lymph nodes	0 = 0; 1 = 1–3; 2 = 4–9; 3 = ≥ 10
PARP1	0 = Low expression, 1 = High expression
Outcome	0 = nonmetastasis; 1 = metastasis

**Table 10:** Predictive role of PARP1 expression in BC metastasis.

Variable	*B*	*SE*	*Wald*	*P*	*RR*	*95% CI*	
PR	− 0.135	0.214	0.399	0.528	0.873	0.574–1.329	
PARP1	1.409	0.257	30.169	<0.001	4.092	2.475–6.766	
Lymph node metastasis							
0	–	–	–	–	–	–	–
1~	0.079	0.343	0.053	0.818	1.082	0.552–2.120	
4~	0.649	0.309	4.424	0.035	1.914	1.045–3.505	
≥ 10	0.849	0.292	8.439	0.004	2.336	1.318–4.142	

#### Predictive effect of NF-κB proteins on distant metastases after BC

##### ROC curve analysis of NF-κB proteins for distant metastasis after BC surgery

**Figure 7:**
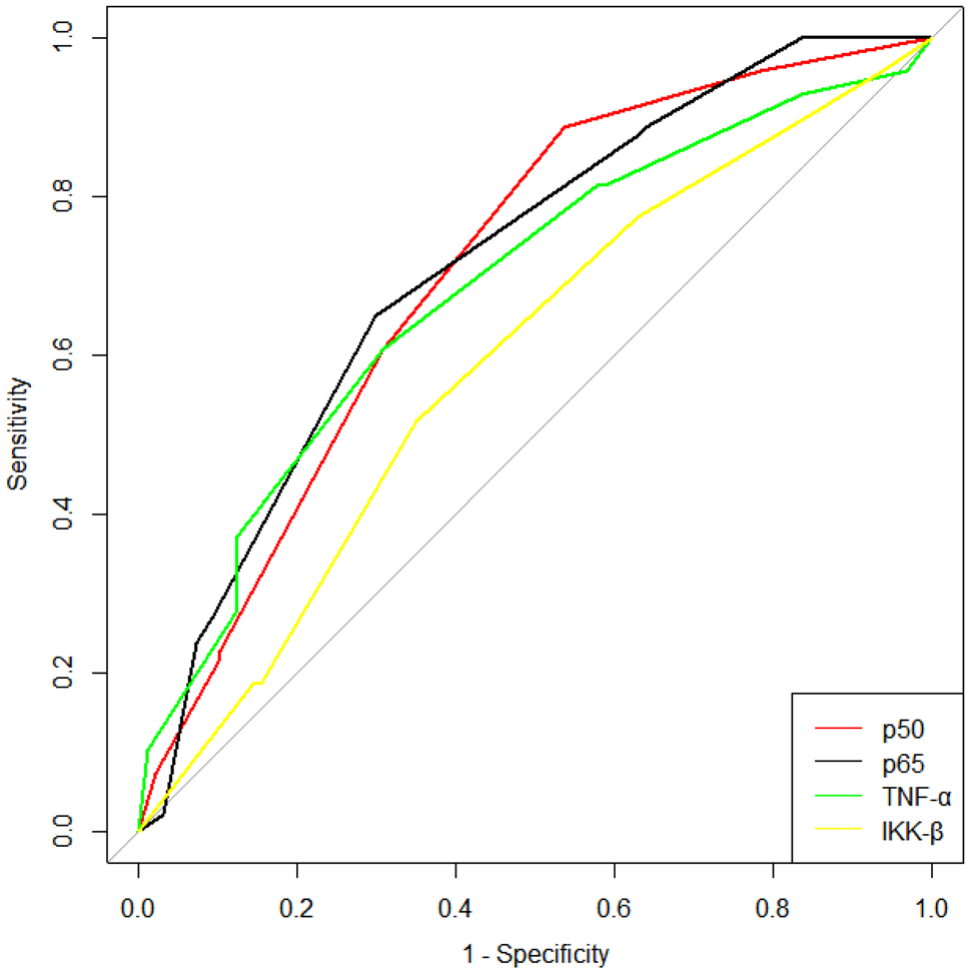
Predictive value of NF-κB proteins for BC metastasis.

ROC curve analysis showed the cut-off values for IKKβ, p65, p50 and TNF-α: for IKKβ, an immunohistochemical score of > 4, *Se* = 51.55%, *Sp* = 64.95%, and *YI* = 0.1649 (*AUC* = 0.591, *P* = 0.025); for p65, an immunohistochemical score of > 4, *Se* = 88.66%, *Sp* = 46.39%, and *YI* = 0.3505 (*AUC* = 0.716, *P* < 0.001); for TNF-α, an immunohistochemical score of > 4, *Se* = 60.82%, *Sp* = 69.07%, and *YI* = 0.2990 (*AUC* = 0.6884, *P* < 0.001); and for p50, an immunohistochemical score of > 2, *Se* = 64.95%, *Sp* = 70.10%, and *YI* = 0.3505 (*AUC* = 0.709, *P* < 0.001). Therefore, they represent the best values for predicting distant metastasis after BC surgery (Fig. 7, Table 11).

**Table 11:** Best diagnostic value of NF-κB proteins.

Indicator	Cut-off value	Sensitivity (%)	Specificity (%)	Youden index	AUC	AUC (95% CI)	
IKKβ	4	51.55	64.95	0.1649	0.591	0.513	0.770
p65	4	88.66	46.39	0.3505	0.716	0.646	0.786
p50	2	64.95	70.10	0.3505	0.709	0.637	0.780
TNF-α	4	60.82	69.07	0.2990	0.6884	0.610	0.758

##### The expression of NF-κB proteins and the relationship between distant metastasis after BC surgery

The immunohistochemical scores for the IKKβ, p65, p50 and TNF-α proteins predicted by ROC curves were used as the grouping basis for their high and low expression, and a chi-square test was conducted according to their respective grouping conditions. The results showed that the high expression levels of p50, p65, TNF-α and IKKβ in the BC metastasis group were significantly higher than those in the nonmetastasis group (*P* < 0.05) (Table 12).

**Table 12:** The expression of NF-κB proteins in BC patients.

Index	Nonmetastasis	Metastasis	$\chi^{2}$	*P*
IKKβ				
Low expression	63 (64.95)	47 (48.45)	5.37	0.02
High expression	34 (35.50)	50 (51.55)		
p65				
Low expression	45 (70.10)	11 (35.05)	29.02	< 0.001
High expression	52 (29.90)	86 (64.95)		
p50				
Low expression	68 (46.40)	34 (11.34)	23.90	< 0.001
High expression	29 (53.60)	63 (88.66)		
TNF-α				
Low expression	67 (69.07)	38 (39.18)	17.45	< 0.001
High expression	30 (30.93)	59 (60.82)		

##### COX regression analysis of the influence of PARP1 protein expression on distant metastasis after BC surgery

According to the cut-off value of TNF-α > 4, the proteins were divided into high- and low-expression groups. After the ROC prediction, the variable distribution is shown in Table 13. Cox regression analysis was performed to verify the predictive effect of TNF-α protein expression on the prognosis of BC. The results showed that the risk of metastasis was 1.825 times higher in the high-expression group than in the low-expression group (*RR* = 1.825, 95%* CI* 1.189–2.799, *P* = 0.006) (Table 14).

**Table 13:** Regression assignment tables.

Variable	Assignment
PR	0 = negative; 1 = positive
Number of lymph nodes	0 = 0; 1 = 1–3; 2 = 4–9; 3 = ≥10
TNF-α	0 = Low expression, 1 = High expression
Outcome	0 = nonmetastasis; 1 = metastasis

**Table 14:** Predictive role of TNF-α expression in BC metastasis.

Variable	*B*	*SE*	*Wald*	*P值*	*RR*	*95% CI*	
PR	− 0.117	0.213	0.303	0.582	0.890	0.587–1.349	
TNF-α	0.601	0.218	7.581	0.006	1.825	1.189–2.799	
Number of lymph nodes							
0	–	–	–	–	–	–	–
1~	0.290	0.337	0.740	0.390	1.336	0.690–2.588	
4~	0.985	0.307	10.280	0.001	2.679	1.467–4.893	
≥ 10	1.113	0.292	14.516	<0.001	3.042	1.716–5.392	

#### Combined prediction of breast cancer metastasis by PARP1 and NF-κB proteins

**Figure 8:**
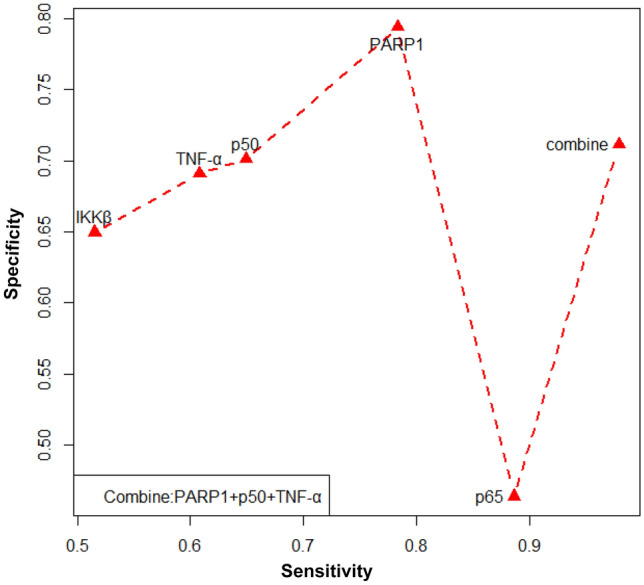
Value of NF-κB proteins in predicting BC metastasis.

Combined with the results of Se, Sp, YI and Fig. 8 above, we can conclude that PARP1, p50 and TNF-α have better predictive effects. There is an intrinsic relationship between the three proteins, and there is a certain synergistic effect between them. Therefore, we combined PARP1, p50 and TNF-α to detect breast cancer prognosis. Joint diagnostic criteria were as follows: high expression of a single index was considered high, and low expression of three indices was considered low (Se = 97.94%, Sp = 71.13%).

## Discussion

BC is a typical inflammatory and highly heterogeneous neoplastic disease with different biological and histopathological features as well as different clinical courses and therapeutic responses. Age, ER, PR, HER2, Ki67, P53 and Ecad are often used to evaluate the prognosis of patients in clinical practice. However, under these traditional diagnostic tools, patients with the same clinicopathological parameters are often likely to show significantly different clinical courses and prognoses. In recent years, the application of endocrine, targeted therapy drugs represented by PARP1 inhibitors and neoadjuvant therapy has made traditional diagnostic indicators unsuitable for predicting the prognosis of BC patients. Therefore, the search for new potential biomarkers for predicting prognosis and metastasis in BC patients is an ongoing task.

### Relationship between the DNA repair gene PARP1 and distant metastasis after BC surgery

In clinical practice, PARP1 inhibitors are frequently employed as therapeutic drugs for BC and other solid tumours since PARP1 is an important DNA repair promoter. It can encourage the emergence and subsequent development of tumours and plays a role in tumour control related to inflammatory mediators. Chemotherapy regardless of hormonal therapy and peritumoral vascular invasion have been found to be substantially linked with the expression of PARP1 in BC. The PARP1 protein is significantly more expressed in BC tissues than in non-BC tissues, according to a meta-analysis of breast malignancy by Goncalves et al.^33^, and it is significantly more expressed in triple-negative BC tissues than in other kinds of BC tissues. These investigations have demonstrated a strong correlation with PARP1 and the prognosis and malignant progression of BC. The PARP1 protein was expressed in the nucleus and cytoplasm of BC cells in this research, with the nucleus being the primary location of the protein as identified by immunohistochemical analysis of BC paraffin blocks. It was discovered that the expression of PARP1 was considerably higher in the BC metastasis group than in the control group, and Spearman correlation analysis revealed a positive link between the expression of PARP1 and BC metastasis. Therefore, the immunohistochemical PARP1 score was analysed via ROC curve assessment to further examine the function of PARP1 in predicting distant metastasis following BC surgery and lessening the impact of discontinuous values. An appropriate cut-off value was chosen to accurately assess PARP1 expression in the two groups of BC patients. The results showed that PARP1 was highly expressed when its immunohistochemical score was > 6 (*Se* = 78.35%, *Sp* = 79.38%, *YI* = 0.5773, *AUC* = 0.843). Finally, Cox regression analysis after grouping showed that the RR value of PARP1 was 4.092, which further proved that the expression of PARP1 is a risk factor for distant metastasis after BC surgery and indicated that when the PARP1 immunohistochemical score is > 6, it can be used to predict the prognosis of BC to a certain extent.

### Relationship between proteins associated with the NF-κB pathway and distant metastasis after BC surgery

IKK/NF-κB regulates the expression of multiple genes in vivo to participate in various biological processes, such as inflammation, apoptosis, and tumour development. Some studies have shown that the activity of NF-κB pathway proteins is indeed related to lymph node metastasis, tumour size, invasion and other clinicopathological characteristics and may contribute to the development of tumour cells that are resistant to chemoradiotherapy. Abnormal expression of p65/p50 can promote the proliferation and differentiation of BC cells and tumour angiogenesis and regulate the transcription of various adhesion- and metastasis-related factors to promote tumour invasion and metastasis^34^. Inhibiting the activity of the NF-κB pathway can increase the susceptibility of those with BC to antitumour medications and endocrine therapy by downregulating the expression of NF-κB pathway proteins in MCF-7 cells^35^. In this study, IKKβ, p65 and p50 proteins of the NF-κB pathway were mainly expressed in the cytoplasm, and a small portion of them were expressed in the nucleus. This study also found that the expression scores of IKKβ, p65 and p50 proteins in the BC metastasis group were significantly higher than those in the nonmetastasis group, and there was a positive correlation between IKKβ, p65 and p50. TNF-α is one of the earliest factors produced in the inflammatory system of BC. It is initiated by NF-κB signalling pathway activation, which works in conjunction with the NF-κB signalling pathway to promote the development and dissemination of BC cells. It can also be utilized to trigger NF-κB pathway activation in tumour cells. TNF-α expression is much higher in BC patients’ serum than in the general population, and it is closely correlated with the TNM stage and invasiveness of tumours^16,17^. Additionally, several studies have discovered that TNF-α expression levels are a prognostic sign for individuals with metastatic BC who will survive without progressing and who will live the longest^18^. The findings of this experimental investigation are consistent with all the studies mentioned above. Additionally, our study demonstrated that the expression of TNF-α was linked to distant metastasis following BC surgery, with a considerably greater expression rate in the metastatic group than in the nonmetastatic group. TNF-α expression and the total number of lymph node metastases were both identified as risk variables for the prognosis of BC metastasis by Cox regression analysis using general clinical pathological data. Regression analysis was consequently carried out after ROC analysis. The final findings demonstrated that TNF-α was a risk factor for predicting the prognosis of BC metastasis and that the chance of postoperative metastasis was 1.825 times higher in the presence of high expression than in the absence of high expression.

### Predictive role of PARP1 and NF-κB pathway proteins in BC metastasis

ROC curve analysis showed that the Se of IKKβ was only 51.55%, and the Sp of p65 was lower than 50%. Therefore, PARP1 > 6, p50 > 2, and TNF-α > 4 had a good predictive effect on postoperative metastasis of breast cancer. IKKβ is tightly associated with DNA repair in cancer cells, and Parp1-mediated NF-κB signalling governs cell ageing. TNF-α is also connected to PARP1^29^. Research^36^ has demonstrated that the inflammatory factor TNF-α, which in turn can activate the PARP1 gene and TNF-α, can directly activate the NF-κB pathway, can be expressed by PARP1 and can maintain the long-term activation of p65 in the NF-κB pathway. Moreover, research on gastric cancer has demonstrated that PARP1 can control tumour growth via the NF-κB pathway^37^. Synergistic transcription between PARP1 and the NF-κB pathway has also been discovered in BC cells. Patients with invasive BC have been reported to have NF-κB transcriptional regulation mediated by PARP1 as a predictive factor^38^. The findings of this study demonstrated a correlation between PARP1 and important elements of the NF-κB pathway. The expression of TNF-α, a downstream component of the NF-κB pathway, was positively linked with PARP1, as was the expression of p65 and p50. The prognosis of BC metastasis was correlated with the expression of PARP1, IKKβ, p65, p50, and TNF-α. This raises the possibility that PARP1 may control TNF-α via the NF-κB signalling pathway to promote BC metastasis, although additional confirmation in cell and animal studies is needed. Therefore, the combined prediction of PARP1, p50 and TNF-α has a good prediction effect, and the prediction sensitivity can be increased to 97.94%. Although the specificity is reduced compared to a single indicator, the clinical significance of increased sensitivity in predicting cancer prognosis far outweighs its specificity.

## Conclusion

The presence of PARP1, IKKβ, p50, p65, and TNF-α is positively associated with distant metastasis, indicating that these factors may be crucial in the spread of BC. Via the NF-κB signalling pathway, PARP1 may control TNF-α’s impact on the metastasis of BC, offering insights into the molecular basis of this process. PARP1 > 6, p50 > 2, and TNF-α > 4 have a certain predictive effect on breast cancer metastasis, and the predictive effect is better when they are combined in diagnosis.

### Limitations and advantages of the study

This study was a prospective nested case‒control study with complete data, which can save considerable manpower, material and financial resources. Exposure data for this study were collected before the disease was diagnosed. This study only preliminarily discussed the predictive role of the DNA repair genes PARP1 and NF-κB pathway proteins on postoperative breast cancer metastasis but has not yet further studied their regulatory mechanisms in breast cancer metastasis and the screening of drug targets. Our team plans to conduct further cell experiments in future research.

## Data Availability

The majority of the study’s data are presented in this paper or in the Supplementary files, and any additional data can be obtained upon reasonable request from the corresponding author.
